# Dissecting of the paravesical space associated with lower urinary tract dysfunction – a rat model

**DOI:** 10.1038/s41598-020-58604-4

**Published:** 2020-02-03

**Authors:** Tsia-Shu Lo, Yi-Hao Lin, Ma. Clarissa Uy-Patrimonio, Hsiao-Chien Chu, Wu-Chiao Hsieh, Sandy Chua

**Affiliations:** 1 Department of Obstetrics and Gynecology, Chang Gung Memorial Hospital, Keelung, Medical Center, Keelung, Taiwan Republic of China; 2 Division of Urogynecology, Department of Obstetrics and Gynecology, Linkou, Chang Gung Memorial Hospital, Linkou Medical Center, Taoyuan, Taiwan Republic of China; 3 Chang Gung University, School of Medicine, Taoyuan, Taiwan Republic of China; 4 Department of Obstetrics and Gynecology, Dr. Pablo O. Torre Memorial Hospital, Bacolod City, Philippines; 5 Fellow of the Division of Urogynaecology, Department of Obstetrics & Gynaecology, Chang Gung Memorial Hospital, Linkou, Taoyuan, Taiwan Republic of China; 6 Department of Obstetrics and Gynecology, Cebu Institue of Medicine- Cebu Velez General Hospital, Cebu City, Philippines

**Keywords:** Membrane structure and assembly, Urological manifestations

## Abstract

To determine the association of opening the paravesical space in relation to its occurrence of *de novo* SUI in an animal model. Thirty five female Sprague Dawley rats were divided into 5 groups of 7 rats each: Control group, Sham groups(F, H), and Study groups(MF, MH). Groups labeled with “F” had the paravesical space opened, “H” had tissue dissection with no opening of the space, and “M” had mesh implanted inside the vaginal wall. Urodynamic studies, immunohistochemical analysis, and western blot were done at day 40. The mean weight and age of 35 rats were 302.1 ± 25.1 grams and 12.8 ± 1.2 weeks old. No significant differences were noted among the control, Sham F, Sham H, Study MF, and Study MH on the voiding pressure and voided volume. The Sham F and Study MF (opened paravesical space) groups had significantly lower values on leak point pressures (LPP) (p = 0.026; p < 0.001) and shorter voiding intervals (p = 0.032; p = 0.005) when compared to other groups. Immunohistochemical analysis showed IL-1 and TNF-α to be intensely increased for the Study MF group (p = 0.003; p = <0.001). MMP-2 and CD 31 markers were also significantly higher in the Study MH and MF group. NGF expression was significantly increased in the Study MF and Sham F groups. Thus, opening of the paravesical space causes an increased inflammatory reaction, which leads to tissue destruction and lower urinary tract dysfunction, exemplified in the study with low leak point pressure and shortened voiding intervals.

## Introduction

Stress urinary incontinence (SUI) has affected adult women with an incidence 4% to 35%^1^. Widely recognized risk factors associated with the development of SUI include aging, obesity, and smoking where as birth trauma and anatomical distortion due to pelvic organ prolapse are somewhat controversial^1^.

Of particular concern nowadays is the occurrence of *de novo* SUI noted with increasing frequency after pelvic organ prolapse corrective surgeries. Wei *et al*.^2^ reports an incidence of 43% after prolapse surgery and Lo *et al*.^3^ demonstrates the occurrence of *de novo* SUI at 26.3% after prolapse surgery using mesh kits such as the Elevate^TM^ system. In a separate study by Lo *et al*.^4^ 23% of patient developed post-operative *de novo* SUI after using the Uphold^TM^ (Boston Scientific) Lite system. The similarity that these two separate studies shares is that, these mesh kits utilizes the anterior approach for bilateral sacrospinous ligament fixation. This procedure entails opening the paravesical space, in which Lo *et al*.^3,4^ in his two studies has postulated that opening this space could lead lowering of the mean urethral closure pressure (MUCP), hence SUI. Moreover, other predictive factors also contribute to the occurrence of *de novo* SUI. These would include age >66 years, diabetes mellitus, maximum urethral closure pressure (MUCP) <60 cm H_2_O and functional urethral length (FUL) <2 cm^5^.

At present, all available data with regards to the occurrence of *de novo* SUI are limited to postulated theories. There have been no reported studies attempting to identify the cause of the occurrence of *de novo* SUI. However, there exists a model for the occurrence of SUI among mice. A study replicated birth trauma in a knockout mouse through vaginal distention. It was proven that vaginal distention induced SUI, with the severity related to the distention. Partial urethral denervation was mentioned as a plausible cause^6^. Since most corrective surgeries use prolene mesh for anterior and apical support, these were also tested on mice. Results of the study showed increased sub-urethral tissue matrix metalloproteinase and nerve growth factor expression that relates to tissue remodeling after prolene mesh implantation for stress urinary incontinence. Based on these studies, replicating SUI was possible in mice which made the authors come up with the idea to replicate corrective prolapse surgeries that open the paravesical space to be able determine its’ association in relation to the occurrence of *de novo* SUI.

## Materials and Methods

This is an experimental study design with approval and funding obtained from the Institutional Animal Care and Use Committee of Chang Gung Memorial Hospital (IACUC No.: 2015070701). The experimental procedures were done in accordance with the relevant guidelines and regulations of the institution. The animals used for the research were female Sprague Dawley rats. These rats were purposely bred for such purpose by Biolasco Taiwan Co., Ltd Taipei, Taiwan. Thirty-five rats were used in the study. These were divided into 5 groups, containing 7 rats in each group. The animals were grouped as follows: Control, Sham H, Sham MH, Study F, and Study MF. The Study F and Study MF underwent opening the paravesical space while Sham H and Sham MH underwent anterior colporrhaphy. Groups with “M” (Sham MH and Study MF) were implanted with mesh.

### Surgical procedure

First, general anesthesia was administered using Isoflurane. Then, cefazolin was given as pre-operative antibiotic prophylaxis. The rat’s vagina was then exposed using a Lone Star retractor system (Cooper Surgical). Hydrodissection was then carried out with the use of normal saline solution injected into the anterior vaginal wall, about 0.5–1.0cc in amount. For the Sham groups, the anterior vaginal wall was dissected laterally (Sham H), and a piece of square mesh measuring 0.5 × 0.5 cm was inserted into the space created (Sham MH). For the Study groups, the space between the vagina and bladder was dissected to open and enter the paravesical space (Study F). In addition, a polypropylene mesh was inserted into the space for the “Study MF”. The vaginal mucosa was then closed with Polyglactin 5-0 suture (Vicryl).

### Conscious cystometrogram measurement

Suprapubic Tube Implantation (SPT) was done as described by Lo *et al*.^7^. Forty eight hours after SPT, the rats were placed in metabolic cages (Med Associates Inc., St. Albans, VT) for 70–80 minutes. The bladder catheter that was previously placed was connected to the syringe pump and amplified pressure transducer. All bladder pressures were referenced to air pressure values at the level of the bladder. The bladder was then filled with 0.9% saline solution at 5 mL/hr. Bladder pressure was recorded. A beaker was placed beneath each cage for urine collection and measurement. Changes in urine weight were recorded as well. Saline infusion was continued until rhythmic bladder micturition contractions became stable. Once stable, data on at least 5 representative micturition cycles were collected for cystometrogram. Voided volume, defined as the volume expelled during micturition was likewise recorded. Peak voiding pressure was measured at the peak of the detrusor contraction. The inter-contraction interval between two successive contractions was calculated in micturition cycles.

### Immunohistochemical analysis

The rats were euthanized by isoflurane overdose immediately after cystometrogram analysis on day 40. The rat’s urethra was removed and was divided into two parts. For the histological examination, the specimen was fixed in a compound of optimal cutting temperature (Sakura Finetek Japan Co., Tokyo, Japan) and quickly frozen at −70 °C until analysis. Coronal sections of 10 µm in thickness were made using a freezing microtome (Leica Biosystems Nussloch GmbH, Nussloch, Germany). The remainder was kept for western blot analysis.

The sample was homogenised in a lysis buffer (PRO-PREPTM solution, iNtRON BIOTECHNOLOGY). The cell lysis was induced by incubation on ice for 20 minutes. The lysis was centrifuged at 13,000 rpm and 4 °C for 10 minutes, and the supernatant was transferred to a fresh 1.5 mL tube. The protein content of the supernatant was estimated by the Bradford method. The samples (30 µg per lane) were mixed with a sample buffer containing 10% mercaptoethanol (Sigma). The mixture was heated at 100 °C for 10 minutes and applied to a 10% sodium dodecyl sulfate polyacrylamide gel for electrophoresis. The protein was electrophoretically transferred into a nylon membrane. Nonspecific binding was blocked for 1 hour at room temperature with 10% (w/v) milk. After washing the membrane with TBS containing 0.1% (v/v) Tween 20 thrice, each of 10 minutes duration, the membrane was incubated overnight at 4 °C with the antibody in 1:1000 dilution. After rinsing, the membrane was incubated with goat anti-rabbit lgG horseradish peroxidase conjugate antibody (SIG-A0545, sigma, 1:10000). The membrane was incubated in the chemiluminescence reagent for 5 minutes and exposed to high performance chemiluminescence film. The film was developed and used to measure the optical density. The optical density of the band was quantified by the UN-SCAN-IT gel TM gel & graph digitizing software.

The outcome measures were the density of inflammatory reaction produced by the IL-1, TNF-α, NGF, MMPs and CD-31 around the surgical site/area of implants and their association with the functional urodynamic studies of the rats at day 40.

### Leak point pressure (LPP)

LPP was measured as previously described by Lin *et al*.^6^. The rats were anesthetized with urethane (1 g/kg). The bladder was emptied manually using Credé's maneuver, and then filled with room-temperature saline at 10 mL/h through the bladder catheter. The average bladder capacity of each rat was determined after three to five voiding cycles. When half-bladder capacity was reached, gentle pressure with one finger was vertically applied to the rat’s abdomen. Pressure was gently increased until urine leaked. The peak bladder pressure at which urine leaked was taken as the LPP. Five measurements were obtained on each animal, and the mean LPP was recorded.

### Statistical analysis

Descriptive statistics were used in the analysis of NGF, IL-1, TNF-α, MMP-2, and CD-31 results. ANOVA and Fisher exact test were applied for comparison of categorical data. When the assumption of the chi-squared test was violated (i.e., when more than one cell had an expected count of <1 or >20% of the cells had an expected count of <5), Fisher’s exact test was used. Values of p < 0.05 were considered statistically significant for all comparisons. All statistical methods were performed using the commercial software SPSS, version 17.

## Results

The mean weight and age of the 35 rats were 302.1 ± 25.1 grams and 12.8 ± 1.2 weeks old, respectively. All rats survived until the end of the experiment, with no complications observed during the post implantation period.

### Cystometrogram and LPP measurement

**Figure 1:**
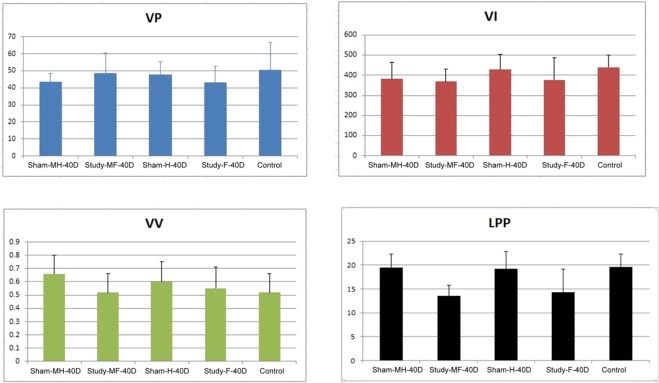
The magnitude of changes in post-operative Urodynamic study VP, VI, VV and LPP at day 40. Legend: VP-Voiding pressure, VI-Voiding Interval, VV-Voiding Volume, LPP-Leak point pressure, MH-paravesical space not opened with mesh implanted, MF-paravesical space opened and mesh implanted, H-paravesical space not opened, F-paravesical space opened.

Day 40 urodynamic study results of SD rats showed no significant change in voiding pressure and voided volume among groups (Table 1). Voiding interval for Study F (377.2 + 108.9 sec) and Study MF (369.4 + 60.8 sec) were significantly shorter (p = 0.32; p = 0.005, respectively) when compared against the control. However, when Sham MH and Study MF were compared, the difference was not significant. Leak point pressure (LPP) values in the Study F (14.3 ± 4.9 μL) and Study MF (13.5 ± 2.3 μL) groups were significantly lower than the control (p = 0.026; p =<0.001). Furthermore, when these values were compared against Sham MH group, the low LPP value of the Study MF remained statistically lower. A graphical representation of the results was presented in Fig. 1.

**Table 1:** Post-operative Urodynamic study (UDS) parameters in control, sham (para-vesical space with and without opened), and study (para-vesical space with and without mesh implanted) groups at Day 40.

VP (cm H_2_O)			p value^a^ (inter group)	p value* (inter group)	p value** (inter group)
n = 7	Normal	50.4 ± 16.2	0.388		
n = 7	Sham-H	47.5 ± 7.8		(reference)	
n = 7	Sham-F	42.8 ± 9.7		0.338	
n = 7	Study-MH	43.5 ± 5.0		0.277	(reference)
n = 7	Study-MF	48.5 ± 11.7		0.327	0.304
**VI (sec)**					
n = 7	Normal	439.6 ± 91.2	0.001		
n = 7	Sham-H	430.0 ± 74.2		(reference)	
n = 7	Sham-F	377.2 ± 108.9		**0.032**	
n = 7	Study-MH	382.2 ± 81.1		0.121	(reference)
n = 7	Study-MF	369.4 ± 60.2		**0.005**	0.273
**VV(μl)**					
n = 7	Normal	0.65 ± 0.19	0.762		
n = 7	Sham-H	0.60 ± 0.15		(reference)	
n = 7	Sham-F	0.55 ± 0.16		0.549	
n = 7	Study-MH	0.66 ± 0.14		0.313	(reference)
n = 7	Study-MF	0.52 ± 0.14		0.211	0.094
**LPP(μl)**					
n = 7	Normal	19.6 ± 2.7	0.001		
n = 7	Sham-H	19.2 ± 3.6		(reference)	
n = 7	Sham-F	14.3 ± 4.9		**0.026**	
n = 7	Study-MH	19.5 ± 1.8		0.604	(reference)
n = 7	Study-MF	13.5 ± 2.3		**<0.001**	**<0.001**

VP, voiding pressure; VV, voiding volume; VI, voiding interval; LPP, leak point pressure. Data listed as mean ± standard deviation with 95% confidence intervals in parentheses.

*Sham-H and Sham-F, Sham-H and Study-MH, Sham-H and Study-MF.

**Study-MH and Study-MF.

Statistical analysis by ^a^ANOVA and Fisher’s exact test.

Data listed as mean ± standard deviation.

### Immunohistochemical and western blot analysis

Immunohistochemical analysis of the mid-urethra at Day 40 (Table 2) showed the pro-inflammatory mediator IL-1 to have increased density for all groups but only the Study MF (81.9 ± 15.1) group had a significant difference over the control (p = 0.003) and the Sham MH group (p = 0.062). Likewise, TNF-α exhibited significant increase in density for the Study-F [79.5 ± 7.0; p = 0.003] and Study-MF group [86.7 ± 5.9; p = <0.001]. However, when density values were compared against Sham MH group, only the Study MF group showed significant difference (p < 0.001).

**Table 2:** Immunohistochemistry analysis of IL-1, TNF-α, MMP-2, NGF and CD 31 in control, sham (para-vesical space with and without opened), and study (para-vesical space with and without mesh implanted) groups at Day 40.

IL-1		Density	p value^a^ (inter group)	p value* (inter group)	p value** (inter group)
n = 7	Normal	44.9 ± 6.0	0.013		
n = 7	Sham-H	48.9 ± 10.4		(reference)	
n = 7	Sham-F	60.7 ± 11.0		0.199	
n = 7	Study-M-H	60.3 ± 12.5		0.300	(reference)
n = 7	Study-M-F	81.9 ± 15.1		**0.003**	**0.062**
**TNF-α**					
n = 7	Normal	37.2 ± 6.1	<0.001		
n = 7	Sham-H	55.1 ± 4.4		(reference)	
n = 7	Sham-F	79.5 ± 7.0		**0.003**	
n = 7	Study-M-H	61.3 ± 4.8		0.086	(reference)
n = 7	Study-M-F	86.7 ± 5.9		**<0.001**	**<0.001**
**MMP-2**					
n = 7	Normal	55.5 ± 4.9	**<**0.001		
n = 7	Sham-H	47.6 ± 3.6		(reference)	
n = 7	Sham-F	51.9 ± 4.8		0.123	
n = 7	Study-M-H	78.2 ± 6.2		**<0.001**	(reference)
n = 7	Study-M-F	92.3 ± 4.4		**<0.001**	**0.024**
**NGF**					
n = 7	Normal	10.4 ± 2.8	0.031		
n = 7	Sham-H	9.1 ± 2.7		(reference)	
n = 7	Sham-F	15.3 ± 2.4		**0.043**	
n = 7	Study-M-H	9.6 ± 2.6		0.800	(reference)
n = 7	Study-M-F	15.2 ± 1.8		**0.018**	**0.015**
**CD31**					
n = 7	Normal	8.7 ± 1.4			
n = 7	Sham-H	9.9 ± 1.7		(reference)	
n = 7	Sham-F	10.9 ± 2.0		0.513	
n = 7	Study-M-H	13.0 ± 2.0		**0.031**	(reference)
n = 7	Study-M-F	14.6 ± 2.1		**0.019**	0.377

*Sham-H and Sham-F, Sham-H and Study-MH, Sham-H and Study-MF.

**Study-MH and Study-MF.

Statistical analysis by ^a^ ANOVA and Fisher’s exact test.

Data listed as mean ± standard deviation.

Differences in the histologic tissue changes in the anterior vaginal wall through expression of MMP-2 and CD31 were observed against the Sham H group instead of control. MMP-2 values for Sham MH (78.2 ± 6.2) and Study MF (92.3 ± 4.4) groups were significantly increased (p = <0.001). Moreover, when both groups were compared against each other, the difference was noted to be significant as well (p = 0.024). CD31 was detected to have a significant increased density in the Sham MH (13 ± 2.0; p = 0.031) and Study MF (14.6 ± 2.1; p = 0.019) groups. However, when compared with Sham MH, the increase observed in Study MF was not statistically significant.

**Figure 2:**
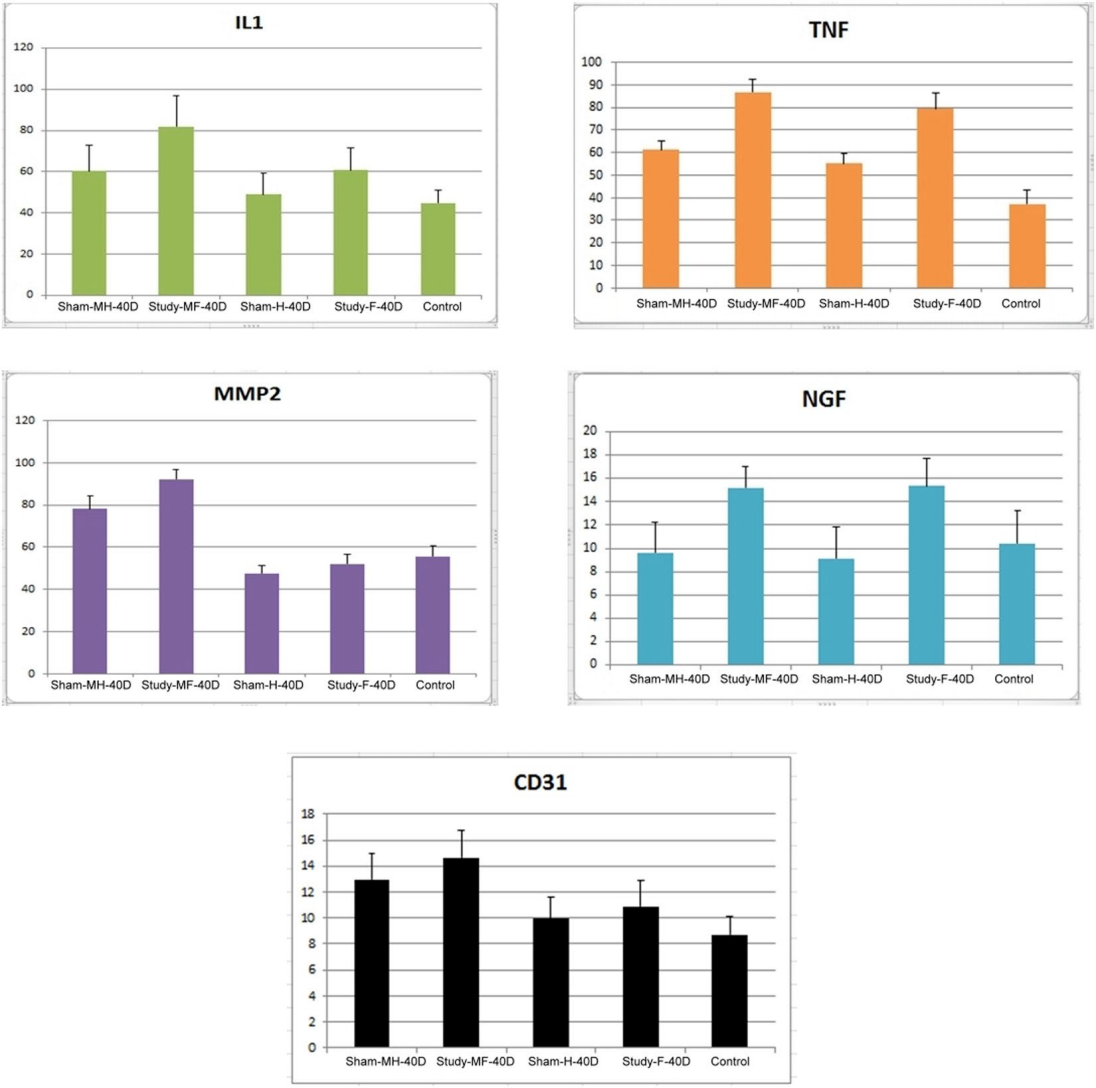
The magnitude of changes in immunohistochemical evaluation of IL-1, TNF-α, MMP-2, NGF and CD31 on Day 40 after surgery in SD rats. Legend: MH-paravesical space not opened with mesh implanted, MF-paravesical space opened and mesh implanted, H-paravesical space not opened, F-paravesical space opened.

**Figure 3:**
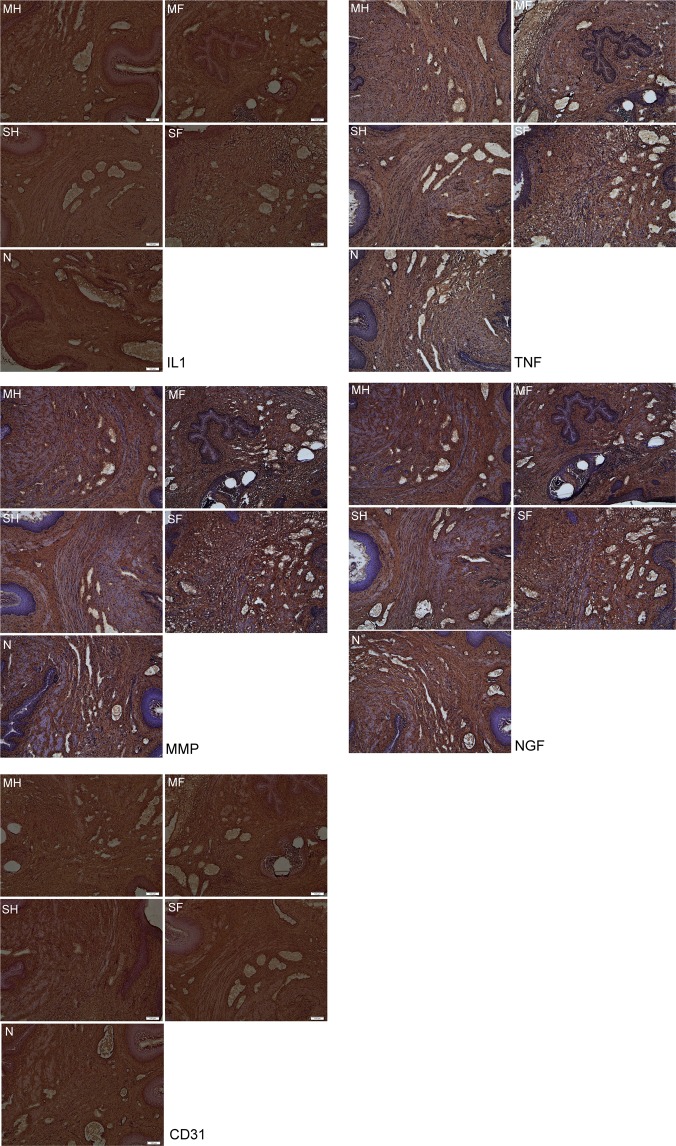
Analysis of immunoreactive expression of IL-1, TNF-α, MMP-2, NGF and CD31at 40 days post-surgery. (x100) Legend: MH-paravesical space not opened with mesh implanted, MF-paravesical space opened and mesh implanted, H-paravesical space not opened, F-paravesical space opened. *Brown spots signifies inflammatory cells (Reagents: anti-NGF/TA300799/origene; anti-IL1 antibody/TA336742/origene; anti-MMP2 antibody/TA336592/origene; anti-TNF antibody/PA5-19810/Thermo; anti-CD31 antibody/PA5-24411/Thermo).

The NGF marker, known to be associated with lower urinary tract symptoms, was observed to have a significant increase in the Study F (15.3 ± 2.4; p = 0.043) and Study MF (15.2 ± 1.8; p = 0.018) groups when compared against the Sham H. In addition, when values were compared against Sham MH, the increase in Study MF levels maintains statistical significance (p = 0.015). Pictures demonstrating density of the markers are demonstrated in Figs. 2 and 3.

## Discussion

The evolving concern on the effect of implanted mesh on the vagina and the occurrence of *de novo* SUI after pelvic reconstructive surgery has been a subject of debate. The cause of such occurrence has been commonly attributed to opening of the paravesical space and the provocative inflammatory effect brought about by the implanted mesh.

The results of the study show absence of significant change in voiding pressure and voided volume amongst all groups. This is a reasonable observation despite having groups implanted with mesh since the mesh was implanted in a tension free manner preventing such changes to occur. This demonstrates the importance of a tension free application of the mesh in the anterior vaginal wall to avoid changes in voiding pressure and volume.

The LPP for Study F and Study MF groups, which had the paravesical space opened, were significantly lower in comparison to control and Sham groups. Several studies have proven the association of low LPP with the occurrence of SUI^3,4,8,9^. The opening of the paravesical space, which is an extensive dissection in the anterior vaginal wall, caused denervation^7,10^ to the external urethral sphincter, resulting in decreased urethral closure ability, leading to low LPP, and eventually, the development of SUI^11^. Bilateral pudendal nerve crash injury or transection has been used in several rat studies to mimic SUI^12,13^.

In addition, opening of the paravesical space causes a paravaginal defect that leads to symptoms of SUI as well. Due to anatomical proximity between the urethra, bladder neck and vagina, a defect in one compartment causes a cascade of lower urinary symptoms. Paravaginal defects cause a loss of support for the bladder and bladder neck that leads to hypermobility of the bladder neck, leading to symptoms of SUI^13^. Bruce RG *et al*.^14^ demonstrates a 79% cure of SUI when the paravaginal defect was repaired.

Moreover, the shortened voiding interval noted in these groups (Sham F and Study MF), were caused by the increased inflammatory reaction brought about by opening of the paravesical space. Implantation of the mesh did not significantly add to the inflammatory reaction since Study MH group and Sham H showed no significant change. In addition, no significant change was observed on the voiding interval between Study MH and Study MF group as well. The gap of differences related to para-vesical opening between Study MH and Study MF group was close up by mesh implant procedure. NGF, a neurotropin that undergoes intracellular proteolytic cleavage can mediate apoptosis^15,16^, for which this particular marker was significantly increased in these groups. The resultant apoptosis contributes to voiding dysfunction, demonstrated as shortened voiding interval. Whether these rats had urgency or increased frequency is hard to differentiate. Despite this, the finding of the current study can correlate to women developing *de novo* urgency or frequency after mesh placement that opened the paravesical space. A couple of studies had demonstrated increased NGF levels on patients with overactive bladder and bladder inflammation^17,18^.

The present study further evaluates inflammatory reaction and tissue remodeling. Inflammatory mediators such as, IL- 1 and TNF-α, were significantly increased in the study MF group that underwent mesh implantation and dissection of the paravesical space. The inflammatory reaction points to further recruitment and activation of other inflammatory cells that drive cells to tissue destruction and apoptosis^19^ forcing lower urinary tract symptoms to appear observed as low LPP and shortened voiding interval in these particular group of rats (Study MF). The findings support clinical practice theories on the association of opening the paravesical and lower urinary tract symptoms.

Matrix metalloproteinase (MMP-2) can cleave extracellular matrix substrates that directly or indirectly activate apoptotic ligands and cytokines. They play a major role in cell proliferation, migration, angiogenesis, apoptosis, and loss of host defense^15,20^. An example of such is CD-31, a marker expressed by inflammatory cells that usually indicates angiogenesis^20^. The present study exhibits significant increase in these markers (MMP-2; CD-31) particular in groups implanted with mesh (Sham MH, and Study MF). This simply illustrates tissue remodeling and delayed wound healing, which is associated with mesh placement. A drawback for such reaction is the occurrence of mesh erosions. Several reported studies have already established the increase in MMP-2 in relation to mesh implantation indicating active tissue remodeling persisting for a long period of time^21^. Clinical practice validates the occurrence of mesh erosion at 3.5%^22^.

The greatest strength of the study is being an experimental study design replicating human conditions under a controlled environment. However, the small sample size and shorter study period limited the study.

## Conclusion

The opening of the paravesical space causes an increased inflammatory reaction, which leads to tissue destruction and lower urinary tract dysfunction, exemplified in the study with low leak point pressure and shortened voiding intervals. This may imply that an extensive procedure can lead to development of *de novo* SUI and OAB-like symptoms of urgency and increased urinary frequency.
